# Supplementary material for: Effects of the visual-feedback-based force platform training with functional electric stimulation on the balance and prevention of falls in older adults: a randomized controlled trial
Source: PeerJ. 2018 Jan 12;6:e4244. doi: 10.7717/peerj.4244 (PMC5768172; doi:10.7717/peerj.4244)
Supplement: Supplemental Information 3 [file peerj-06-4244-s003.docx]

Effects of the visual-feedback-based force platform training with functional electric stimulation on the balance and prevention of falls in elder adults

Study Protocol

1.Background

Prevention of falls among older adults is a crucial public health challenge. Balance training with visual feedback enhances sensorimotor integration through a recalibration of the sensory systems contributing to postural control. Functional electrical stimulation has been used to improve balance in adults with stroke and was recently shown some effects on balance in older adults. Force platform training with functional electric stimulation aimed at improving balance may be effective in fall prevention for older adults.

2.Objectives of Study

To evaluate the effects of the visual-feedback-based force platform balance training with functional electric stimulation on balance and fall prevention in older adults.

3.Inclusion criteria

(1) age 60 and older; (2) ability to walk with or without an assistive device for a minimum of 20 meters; (3) ability to see visual feedback from a computer screen; and (4) ability to follow instructions for testing and training (Mini-Mental State Examination > 23 points)

Exclusion criteria

(1) neurological disorders; (2) severe psychiatric disorders (Hamilton Depression Scale, HAMD, > 21 point, Hamilton Anxiety Scale, HAMA, > 20 point); (3) severe musculoskeletal impairment; (4) terminal cardiovascular problems; and (5) participation in other balance training.

4.Sample size

Power calculations indicated that a sample size of 120 (60 in each group) would have 80% power to detect a between-group difference of 20% in the fall rates after intervention with a significance level of 0.05 and a dropout rate of 10%.

5.Methods/design

Randomized parallel controlled trial

A physician who does not participate in the research process carry out random grouping by using computer-generated random numbers. The sequence was concealed until group assignment and baseline measurement was completed.

Participants were instructed to stand on the force platform. First, participants were told that the feedback line represented the anterior-posterior position of their body and were instructed to keep the line as close to the midline as possible and not exceed the upper and lower boundary lines for 3 min. Then, participants were asked to keep the line as close to the midline as possible and to stay in the two vertical boundary lines for 3 min.

After the force platform training, participants were instructed to performed the functional electrical stimulation training in sitting position, with the ankle, knee, and hip joints at 90˚. First, participants were asked to do the ankle dorsiflexion and received electrical stimulation by the device for 3 min (10s on and 10s off, rectangular-wave pulsed currents, 50 Hz). Then, participants were received electrical stimulation when they did the ankle plantarflexion in 3 min.

In addition, both groups performed the one-leg standing balance exercise for 12 min per day. The exercise were provided 15 days a month for 3 months.

Participants using daily fall diaries recorded the number of falls from the day training started until the follow-up ended.

6. Outcomes

Primary outcome: Fall rates.

Secondary outcomes: The number of injurious fallers and the number of fallers, COP-based balance parameters, The Berg Balance Scale, The Falls Efficacy Scale-International, the Barthel Index.

7. Data analysis

Independent-sample t-tests, Chi-square tests, 2-factor repeated-measures analysis of variance, Post hoc Bonferroni tests
